# Brassinosteroid Signaling Dynamics: Ubiquitination-Dependent Regulation of Core Signaling Components

**DOI:** 10.3390/ijms26104502

**Published:** 2025-05-08

**Authors:** Riguang Qiu, Yan Zhou, Juan Mao

**Affiliations:** 1 State Key Laboratory for Conservation and Utilization of Subtropical Agro-Bioresources, College of Forestry and Landscape Architecture, South China Agricultural University, Guangzhou 510642, China; qiuriguang@163.com (R.Q.); ia536715@stu.scau.edu.cn (Y.Z.); 2 Guangdong Key Laboratory for Innovative Development and Utilization of Forest Plant Germplasm, College of Forestry and Landscape Architecture, South China Agricultural University, Guangzhou 510642, China

**Keywords:** brassinosteroids, ubiquitination, BRI1, BIN2, BES1

## Abstract

Brassinosteroids (BRs) are essential phytohormones that orchestrate various stages of plant growth and development. The BR signaling cascade is mediated through a phosphorylation network involving sequential activation of the plasma membrane-localized receptor kinase Brassinosteroid-Insensitive 1 (BRI1), the cytoplasmic kinase Brassinosteroid-Insensitive 2 (BIN2), and the transcription factors BRI1-EMS suppressor 1 (BES1) and Brassinazole-Resistant 1 (BZR1). These transcription factors activate thousands of nuclear genes. Recent evidence highlights that ubiquitination has emerged as an equally pivotal mechanism that dynamically controls the BR signaling pathway by modulating the activity, subcellular localization, and protein stability of these core signaling components. In this review, we systematically analyze the central role of ubiquitination in determining the function, localization, and degradation of these proteins to fine-tune the outputs of BR signaling. We provide comparative perspectives on the functional conservation and divergence of ubiquitin-related regulatory components in the model plant *Arabidopsis* versus other plant species. Furthermore, we critically evaluate current knowledge gaps in the ubiquitin-mediated spatiotemporal control of BR signaling, offering insights into potential research directions to elucidate this sophisticated regulatory network.

## 1. Introduction

**Figure 1:**
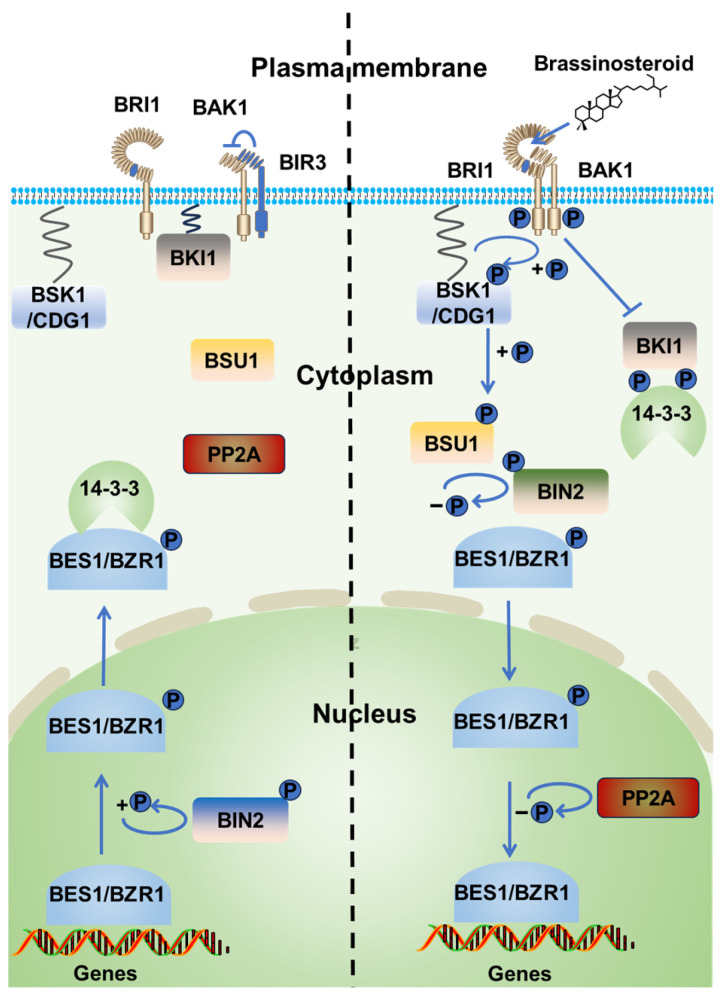
Overview of the BR signaling pathway. When the BR signal is absent (**left**), the receptor BRI1 located on the cell membrane is inhibited by BKI1, and the co-receptor BAK1 is inhibited by BIR3. In the cytoplasm, the kinase BIN2 phosphorylates the transcription factors BES1/BZR1, inhibiting their transcription factor activity and nuclear localization; 14-3-3 binds to BES1/BZR1 in the cytoplasm, inhibiting their entry into the nucleus. When the BR signal is present (**right**), BRI1 and BAK1 activate and form the BRI1-BR-BAK1 complex, relieving the inhibition by BIR3 and BKI1. BSK1/CDG1 is phosphorylated and activates the phosphatase BSU1 to dephosphorylate BIN2, inhibiting its activity. BKI1 phosphorylates 14-3-3 to inhibit its function. The uninhibited BES1/BZR1 is dephosphorylated by PP2A in the nucleus, allowing them to function as transcription factors and regulate downstream gene expression. In the figure, “P” represents phosphorylation.

Brassinosteroids (BRs), as unique plant-specific steroid hormones, are pivotal in orchestrating plant growth and developmental processes [1,2,3]. They also play an essential role in plant adaptability to a variety of biotic and abiotic stresses [4,5]. Extensive research over the past few decades has elucidated BR signal transduction mechanisms, uncovering their regulatory roles in coordinating plant growth and development [6,7] (Figure 1). Current models propose that the perception of BRs occurs through the extracellular domains of leucine-rich repeat (LRR) receptor-like kinase proteins, specifically the Brassinosteroid-Insensitive 1 (BRI1) receptor and its co-receptor BRI1-Associated Receptor Kinase 1 (BAK1) [8,9,10]. Upon BR binding, the BRI1-BAK1 heterodimer undergoes conformational changes that first trigger phosphorylation-mediated dissociation of the inhibitory protein BRI1 Kinase Inhibitor 1 (BKI1) from the plasma membrane, and then undergoes reciprocal trans-autophosphorylation to fully activate the receptor complex [9,11,12,13]. This activation enables the recruitment of downstream receptor-like cytoplasmic kinases (RLCKs), including BR Signaling Kinases (BSKs) and Constitutive Differential Growth (CDG) proteins, which amplify the signal through kinase cascades [14,15]. BSKs/CDGs further phosphorylate and activate BSU1/BSL1-3 phosphatases, leading to dephosphorylation of the GSK3-like kinase BIN2 at its activation loop, thereby inactivating its kinase activity [15,16]. The suppression of BIN2 permits nuclear-localized PP2A phosphatases to dephosphorylate transcription factors bri1-EMS suppressor 1 (BES1) and Brassinazole-Resistant 1 (BZR1) at specific residues, allowing their nuclear accumulation [17,18,19]. Ultimately, dephosphorylated BZR1 and BES1 regulate the expression of BR-responsive genes critical for plant growth and development [20,21].

Beyond the well-characterized phosphorylation–dephosphorylation circuitry, ubiquitination plays a crucial role in precisely tuning the BR signaling amplitude [22,23]. Ubiquitination is an enzymatic cascade process in which ubiquitin molecules (a 78 amino acids protein) are covalently conjugated to substrates through sequential actions of three classes of ubiquitination enzymes [24], including ubiquitin-activating enzyme (E1), ubiquitin conjugation enzyme (E2), and ubiquitin ligase (E3) [25]. The ubiquitination cascade initiates with the ATP-dependent adenylation of ubiquitin’s C-terminal glycine by E1 ubiquitin-activating enzymes, forming a high-energy acyl adenylate intermediate [26]. This activated ubiquitin is subsequently transferred to the catalytic cysteine residue of E2 via a thioester bond [26]. Finally, E3 ligase transfers ubiquitin from E2 to the substrate by forming an isopeptide bond [27]. Based on structural features and catalytic mechanisms, E3 ligases are systematically classified into five major families: homologous to the E6AP carboxyl terminus (HECT), Really Interesting New Gene (RING), Plant U-box (PUB), cullin-RING ligases (CRLs), and Anaphase-Promoting Complex/Cyclosome (APC/C) [24,27].

**Figure 2:**
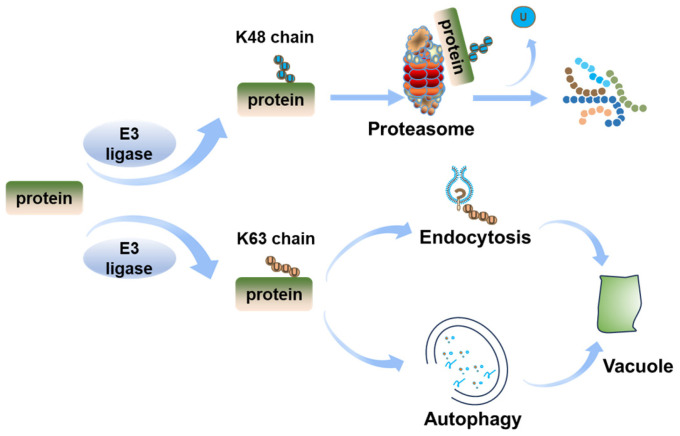
A current model of ubiquitin-dependent plant protein degradation. After being ubiquitinated by E3 ligases, proteins undergo different fates depending on the type of ubiquitin chains formed. Proteins forming K48 chains are degraded by the proteasome. Proteins forming K63 chains are degraded by the vacuole through two different pathways. Membrane proteins undergo endocytosis, forming vesicles that are transported in the cytoplasm, eventually entering the vacuole. Non-membrane proteins form autophagosomes and are transported into the vacuole.

Ubiquitination modifies substrates through two principal mechanisms: monoubiquitination and polyubiquitination. Monoubiquitination involves attaching a single ubiquitin molecule to regulate protein activity and intracellular trafficking [28]. Polyubiquitination promotes the formation of polyubiquitin chains, with eight distinct chain types (K6, K11, K27, K29, K33, K48, K63, M1) identified [28]. Among these different ubiquitination chain forms, the most common forms are K48 and K63 [28] (Figure 2). K48 usually serves as a signal that the protein enters a ubiquitin–proteasome system (UPS) for degradation [29]. On the other hand, proteins modified by K63 polyubiquitin chains mediate protein endocytosis trafficking and vesicle transport [30,31]. K63-linked ubiquitination directs membrane proteins to vacuoles through endocytosis and non-membrane proteins via autophagy [32,33]. Deubiquitinating enzymes (DUBs) comprise five specialized enzyme families, which classify based on their distinct catalytic domains [34]. These enzymes catalyze the removal of ubiquitin by cleaving ubiquitin–substrate linkages, thereby dynamically modulating ubiquitination homeostasis and fine-tuning signaling efficiency through interactions with ubiquitination machinery components [35].

Recent evidence has identified multiple components of the ubiquitination machinery that regulate the functional dynamics of core BR signaling components, including the receptor kinase BRI1, the negative regulatory kinase BIN2, and the transcription factors BES1/BZR1, as well as their upstream ubiquitination regulators (Table 1). In this comprehensive review, we systematically analyze how ubiquitination modulates three critical aspects of BR signaling proteins: (1) functional regulation, (2) subcellular partitioning, and (3) proteostasis control. Additionally, we compare the evolutionary conservation and divergence of ubiquitin-mediated degradation machinery involved in BR signal transduction between the model plant *Arabidopsis thaliana* and other plant species. We also identify key unresolved questions on the regulation of BR signaling by ubiquitination-dependent mechanisms and suggest future research directions to unravel this complex regulatory network.

## 2. BRI1 Regulation by Ubiquitination

As an essential LRR-RLK for sensing the BR signal on the PM, BRI1 exerts strict control over plant growth through spatiotemporal regulation of its PM abundance [65,66]. Overexpression of *BRI1* in *Arabidopsis* promotes plant growth with elongated petioles whereas BRI1 deficiency leads to dwarfism with shorter petioles [65,67]. In other plant species, the expression level of BRI1 shows a significant positive correlation with the rate of biomass accumulation [68,69]. The dynamic equilibrium of BRI1 at the PM is maintained by the delivery of newly synthesized BRI1 protein via the normal secretory pathway, as well as BR-dependent and BR-independent endocytosis that leads to vacuole-mediated proteolysis and recycling back to the PM. Additionally, cytosolic proteasomal degradation further contributes to BRI1 quality control [65,66]. The endocytosis of PM-localized proteins is well known to be regulated by ubiquitination [70]. Therefore, the ubiquitination of BRI1 plays a critical role in controlling the PM abundance of BRI1, thereby governing the activation threshold of the BR signaling process.

### 2.1. Ubiquitination Regulates BRI1 Endocytosis and Degradation

Since identifying BRI1 as a vertical receptor in BR signal transduction, the spatial regulation of BRI1 membrane localization has attracted significant scientific attention. Early studies used overexpression of BRI1-GFP to wild-type levels in BRI1 knockout mutants to mimic endogenous expression patterns, revealing that BRI1 exhibits dual localization on the plasma membrane and intracellular compartments [65]. These studies further established that BRI1 endocytosis occurs constitutively independent of BR signals [65]. Notably, the molecular determinants controlling BRI1 endocytic trafficking remained elusive until Martins et al. found that BRI1 undergoes K63-linked polyubiquitination, mediating its endocytosis [71]. The study also found that BRI1’s kinase activity plays a crucial role in its ubiquitination and endocytosis processes. Among the 29 lysine residues present in BRI1, mutations in 25 lysine residues outside the kinase domain resulted in partial attenuation of BRI1 ubiquitination [71]. Strikingly, mutations targeting the four lysine residues within the kinase domain led to substantially decreased ubiquitination levels and severely impaired endocytosis [71]. However, this paradigm has been challenged by recent studies suggesting that ubiquitination may not serve as an endocytic signal [72]. For instance, researchers screened and mutated multiple lysine residues on BRI1, significantly inhibiting its ubiquitination, but found that its endocytic function was largely unaffected [72]. Although these findings appear consistent with the alternative hypothesis, the potential confounding effects of multi-site mutations on BRI1’s three-dimensional conformation necessitate cautious interpretation. Therefore, comprehensive investigations combining structural analyses and functional assays are required to elucidate the intricate regulatory interplay between BRI1 ubiquitination and membrane trafficking dynamics.

**Figure 3:**
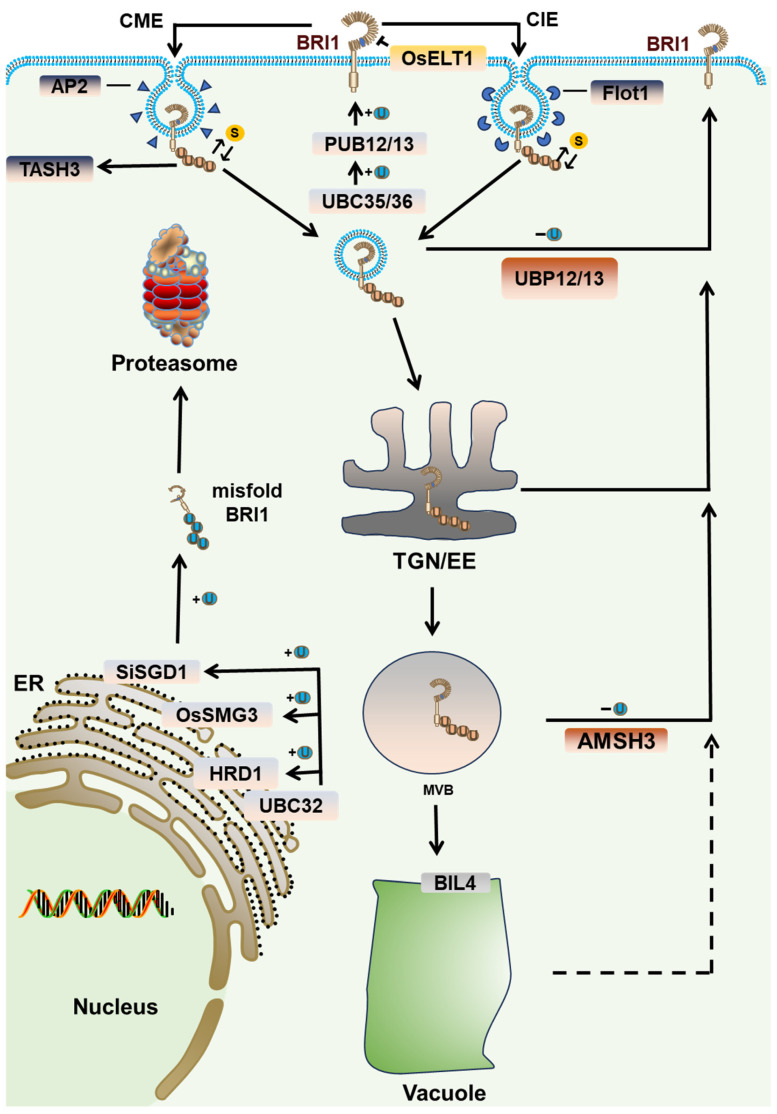
Overview of the ubiquitin-dependent BRI1 endocytosis and degradation. The ubiquitin molecules are transferred by ubiquitin-conjugating enzymes UBC35/36 to the E3 ligases PUB12/13, which ubiquitinate the cell membrane-localized BRI1 to form K63-linked ubiquitin chains. ELT1, which is also localized to the cell membrane, can inhibit the ubiquitination of BRI1. Ubiquitinated BRI1 is recognized by TASH3 and internalized through the CME pathway, although it can also be internalized via the CIE pathway. The vesicles formed by endocytosis are transmitted to the TGN/EE, and finally through MVB enter the vacuole for degradation. During the endocytosis and transmission process, deubiquitinating enzymes UBP12/13 and AMSH3 can remove the ubiquitination from BRI1, allowing it to return to the cell membrane via vesicles. BIL4, localized on the vacuole membrane, also serves as a recognition protein for ubiquitinated BRI1 entering the vacuole. Misfolded BRI1 synthesized in the endoplasmic reticulum is recognized by the Hrd1 complex within the ERAD system, where ubiquitin molecules are transferred by ubiquitin-conjugating enzyme UBC32 to E3 ligase HRD1, leading to K48 chain and proteasomal degradation in the cytoplasm. OsSMG3 and SiSGD1 can also utilize the ubiquitin molecules transferred by the UBC32 homologs OsDGS1 and SiUBC32, resulting in BRI1 ubiquitination and proteasomal degradation.

The ubiquitination of BRI1 is orchestrated through coordinated enzymatic regulation (Figure 3). While Plant U-BOX family ubiquitin ligases PUB12/13 mediate ubiquitin binding to BRI1 in a kinase activity-dependent manner [23], confocal microscopy reveals that residual BRI1-BAK1 oligomers on the membrane maintain sufficient kinase activity to phosphorylate PUB13 [66]. This explains why quantitative imaging analyses show comparable ubiquitination levels of BRI1 under both BR-sufficient and BR-depleted conditions [23,73]. Current research has discovered that UBC35/36 acts as a ubiquitin-conjugating enzyme in the BRI1 ubiquitination process, and the absence of UBC35/36 significantly reduces BRI1 endocytosis, further emphasizing that BRI1 endocytosis relies on ubiquitination [36]. Notably, the reversibility of this modification is governed by deubiquitinating enzymes UBP12/13, which catalyze the cleavage of ubiquitin chains from plasma membrane-localized BRI1, thereby antagonizing its ubiquitination [37]. However, the precise antagonistic regulatory mechanisms between BRI1 ubiquitination and deubiquitination remain poorly defined. BRI1 ubiquitination is regulated by multiple environmental and biochemical factors. Temperature affects BRI1 ubiquitination [74]. At low temperatures, SUMO (a small ubiquitin-like modifier) competes for BRI1 ubiquitin-binding sites, reducing its ubiquitination [22]. In contrast, elevated temperatures promote deSUMOylation, leading to increased BRI1 ubiquitination [22,74]. In rice, the Enhanced Leaf Inclination and Tiller Number 1 (ELT1), which is located on the cell membrane, has been demonstrated to functionally suppress BRI1 ubiquitination [38]. This suggests the existence of membrane-resident regulatory components that may orchestrate BRI1 modification states in plants through analogous mechanisms.

Following endocytic internalization, ubiquitinated BRI1 undergoes vesicular trafficking to the trans-Golgi network/early endosome (TGN/EE) compartment [65]. A subset of these vesicles are sorted through multivesicular bodies (MVBs) for terminal vacuolar degradation, while others undergo recycling back to the cell membrane [65]. Notably, ubiquitination can act not only as a signal for endocytosis but also as an important sorting determinant throughout vesicular transport cascades [75]. Current models propose two mechanistically distinct endocytic routes for BRI1: the constitutive clathrin-mediated endocytosis (CME) pathway and the induced clathrin-independent endocytosis (CIE) pathway [76,77]. In animals, the CME pathway recognizes ubiquitinated membrane proteins through Epsin N-Terminal Homology (ENTH) proteins, but plant ENTH homologs lack canonical ubiquitin-binding domains [78,79,80,81]. Remarkably, plants have evolved a unique compensatory mechanism whereby the TSAUCER (TASH3) subunit of the TPLATE complex (TPC) enables SH3 domain-mediated ubiquitin recognition, thereby facilitating CME-dependent internalization of ubiquitinated BRI1 [41,82]. The molecular mechanism by which the CIE pathway recognizes ubiquitin signals remains elusive, despite the established process where BL-induced Flotillin 1 (Flot1) mediates the invagination [77]. During vesicle trafficking toward the TGN/EE, the cytoplasmic linker protein-associated protein (CLASP) acts as a transport auxiliary protein, redirecting a subset of BRI1 back to the cell membrane [83]. Given that the plant CLASP protein lacks the canonical ubiquitin-binding domains required for signal recognition, there may be other unidentified co-factors involved in the retrograde transport [41]. Following TGN/EE arrival, BRI1 undergoes sequential sorting by the distinct endosomal sorting complex required for transport (ESCRT) machinery components, which ultimately determine its fate between vacuolar degradation and membrane recycling via MVB formation [84]. The sorting of the ESCRT complex requires ubiquitination as a signal, and several proteins are involved in recognizing endocytosed ubiquitinated proteins in the plant ESCRT pathway [85]. Among them, apoptosis-linked gene 2-interacting protein X (ALIX) was found to directly mediate vacuolar targeting of the BRI1 receptor through transport recognition [42]. This process requires the deubiquitination enzyme AMSH3 to remove the ubiquitin chain from the target protein [86]. A recent report pointed out that UBP12/13 can also regulate the ubiquitination status of BRI1 at both plasma membrane and endosomal compartments, but the regulatory relationship between these two deubiquitinating enzymes and BRI1 is not yet clear [37]. Brassinazole-Insensitive-Long hypocotyl 4 (BIL4) localizes to the vacuolar membrane and functions as a gatekeeper of vacuolar degradation—*bil4* mutants display accelerated BRI1 turnover [43]. This suggests that BIL4 affects the vacuolar recognition events of BRI1, but whether ubiquitin acts as a recognition signal requires further validation through in situ ubiquitination mapping and compartment-specific proteomic analyses.

### 2.2. Ubiquitination Regulates BRI1 Degradation by UPS

Although ubiquitinated BRI1 is widely believed to undergo endocytosis and be transported to vacuoles for degradation via vesicle transport, emerging evidence suggests the potential existence of alternative degradation pathways (Figure 3). In animal cells, many membrane proteins undergo ubiquitination with differential fate determination—K63-linked chains typically direct endosome-to-vacuole sorting, whereas K48-linked chains mark substrates for proteasomal degradation [87,88,89]. Studies in vitro have shown that PUB13 catalyzes BRI1 ubiquitination with both K48 and K63 linkages, though in vivo, it predominantly results in K63 chains [36]. Moreover, genetic evidence reveals residual BRI1 ubiquitination persists in *pub12/pub13* mutants, indicating that the type of ubiquitin chain formed by PUB12/13 on BRI1 is regulated by additional factors yet to be identified [23,37,71]. It is hypothesized that other E3 ligases might form K48-linked chains or introduce branched ubiquitin chains on BRI1, thereby directing its degradation by the proteasome [89]. MG132 remains a standard experimental tool in studies investigating BRI1-associated ubiquitin ligases (e.g., PUB12/13) and deubiquitinating enzymes (e.g., UBP12/13) [23,38]. The inhibition of the proteasome with MG132 consistently induces a pronounced accumulation of BRI1 protein across multiple experimental systems, suggesting that ubiquitinated BRI1 can potentially be degraded via the UPS pathway [90,91,92].

The methodological paradigm inadvertently obscures the potential role of UPS in BRI1 degradation [23,37]. During BRI1 synthesis, misfolded proteins are ubiquitinated by UBC32 and E3 ligase HRD1, subsequently transported from the endoplasmic reticulum (ER) to the cytoplasm, and degraded via the ER-associated degradation (ERAD) system [90,93]. Recent studies in rice and foxtail millet report that UBC32 can transfer ubiquitin molecules to decreased grain size 1 (DGS1) and small grain and dwarf 1 (SGD1), two RING family ubiquitin ligases, facilitating BRI1 ubiquitination and regulating the seed size [39,40]. As an integral component of the HRD1 complex involved in ERAD, UBC32 directs ubiquitinated substrates toward proteasomal degradation [93]. This provides mechanistic evidence supporting BRI1’s post-translational regulation through UPS [90,93]. Interestingly, although both studies highlight the UBC32-mediated ubiquitination of BRI1 by these ligases, their proposed mechanisms of action exhibit notable discrepancies. OsDGS1 is proposed to target newly synthesized, misfolded BRI1 in the ER for degradation via the ERAD pathway [39]. Conversely, SiSGD1 appears to regulate BRI1 vesicle transport, facilitating its recycling back to the plasma membrane via the TGN/EE [40]. These findings underscore the complexity of ubiquitination in regulating protein stability. The lack of direct experimental evidence regarding the specific ubiquitin chain linkages formed by these ligases precludes definitive conclusions regarding the degradation pathways for BRI1 homologous proteins in rice and millet. Future studies should employ proteasome loss-of-function mutants to monitor potential changes in BRI1 protein accumulation patterns.

It is crucial to recognize that current analyses of the mechanisms regulating BRI1 ubiquitination, endocytosis, and degradation predominantly depend on live-cell imaging and biochemical analysis of BRI1-GFP fusion proteins. However, this methodology is subject to two significant limitations. Firstly, the expression levels of BRI1-GFP are susceptible to artificial manipulation. Overexpression in transgenic plants often results in protein concentrations substantially higher than the physiological levels of the endogenous protein. This may activate the ER’s quality control system, causing either aberrant retention or enhanced degradation of the fusion proteins, thus obscuring the true characteristics of endogenous ubiquitination and endocytosis pathways [94]. Furthermore, BRI1 functions as a receptor kinase that requires precise conformational changes during its interaction with BAK1. The GFP fusion tag may hinder domain folding or complex assembly, impacting ubiquitination or BR signal transduction. To exclude confounding effects from differential expression profiles, early studies screened genetic materials with BRI1-GFP expression levels that closely matched the endogenous levels and phenotypes comparable to those of wild types [65]. While recent ubiquitination studies often employ a BRI1-GFP complementation system in *bri1* mutants, this approach carries inherent limitations [23,37,71,72]. Although effective in restoring BR responses phenotypically, this approach fails to address a fundamental issue: the GFP tag may alter BRI1’s natural conformation, and heterologous promoter-driven expression may introduce spatiotemporal specificity biases. To overcome these limitations, future studies should directly analyze the ubiquitination profile of endogenous BRI1 by isolating pure plasma membranes and endosomes, combined with quantitative mass spectrometry to avoid interference from fusion proteins. Alternatively, conformation-sensitive probes could be developed to minimize structural disruption and accurately capture dynamic ubiquitination changes in BRI1.

## 3. BIN2 Regulation by Ubiquitination

BIN2 is an important serine/threonine protein kinase that negatively regulates BR signal transduction by phosphorylating the transcription factors BES1/BZR1, thereby inhibiting their nuclear localization and DNA-binding abilities [16,95,96,97]. Enhanced BR signaling promotes the dephosphorylation of BIN2 by BSU1, consequently alleviating its inhibitory effect on downstream signaling components [15,16]. Unexpectedly, overexpression experiments in *Arabidopsis* and rice revealed that increasing the abundance of wild-type BIN2 protein had little impact on the plant phenotype, while elevating the abundance of the gain-of-function mutant protein bin2-1 resulted in pronounced phenotypic alterations, including dwarfism and shortened leaf petioles [98,99]. These observations indicate a complex relationship between the abundance and activity of the BIN2 protein. As ubiquitination directly regulates protein abundance, elucidating the regulatory mechanism of ubiquitination on BIN2 will contribute to a deeper understanding of its functional regulation.

In *Arabidopsis*, the F-box E3 ubiquitin ligase, Kink suppressed in *bzr1-1D* 1 (KIB1), was found to ubiquitinate BIN2 and mediate its degradation via the UPS pathway [44]. Intriguingly, KIB1 specifically ubiquitinates dephosphorylated BIN2 in the cytoplasm, but not bin2-1 (located in nuclear) [44]. However, the mechanism underlying this selective recognition remains unclear. Given that KIB1 is an F-box family ligase that primarily regulates substrate ubiquitination by forming a complex with Cullin 1 (CUL1) [100], it is plausible that a component within this complex possesses the ability to recognize the phosphorylation status of the substrate. In a parallel investigation, two F-box family ligases, BRASSINOSTEROID F-Box Protein 1 (BRFP1) and BRFP2, which interact with BIN2, were identified through IP-MS screening [47,100]. These ligases were found to influence the protein stability of BIN2 [46,101], although conclusive evidence demonstrating direct ubiquitination of BIN2 by these enzymes remains to be established. Additionally, the ubiquitin ligase Constitutively Photomorphogenic 1/Suppressor of phyA-105 (COP1/SPA1) was shown to inhibit the interaction between BIN2 and the transcription factor, Photoperiod-Interactive Factor 3 (PIF3), in the light signaling pathway, without affecting BIN2 protein stability [102]. Thus, it is speculated that COP1/SPA1 may mediate monoubiquitination of BIN2 [103], potentially inducing conformational changes that impair its binding capacity with PIF3.

In rice, the homologous protein of BIN2, OsGSK2, functions as a negative regulator of BR signaling transduction that coordinately controls plant height and grain development [104]. OsGSK2 also influences other hormone signaling pathways and agronomic traits [104]. The U-BOX family ubiquitin ligase, Taihu Dwarf1 (TUD1), ubiquitinates GSK2 and promotes its degradation, impacting BR signaling in rice [45]. Conversely, OsKIB1 does not degrade OsGSK2 [105]. Interestingly, another intriguing phenomenon is observed in rice, where the interaction between the protein phosphatase Kelch-Like 1 (OsPPKL1) and OsGSK3 leads to the dephosphorylation of OsGSK3 and consequent increased protein stability in the cytoplasm [106]. In humans, the BIN2 orthologs GSK3α/β catalyze the inhibitory phosphorylation of β-catenin [107], functionally mirroring BIN2-mediated suppression of BES1/BZR1 in plants. This mechanism holds significance in the pathogenesis of various diseases and as a potential drug target [108]. Further reinforcing the conservation of ubiquitination-dependent control, recent research has revealed that dephosphorylated GSK3β is ubiquitinated by WD repeat and SOCS box-containing protein 1 (WSB1) of the SOCS family and degraded via the proteasome pathway, thereby alleviating the inhibitory effect of GSK3β on β-catenin [109]. BIN2 ubiquitination is dynamically regulated by environmental signals [110]. Together, these findings collectively highlight the evolutionary conservation and species-specific diversification of ubiquitination-mediated regulatory mechanisms governing BIN2 homologous proteins across different species [111].

## 4. BES1/BZR1 Regulation by Ubiquitination

**Figure 4:**
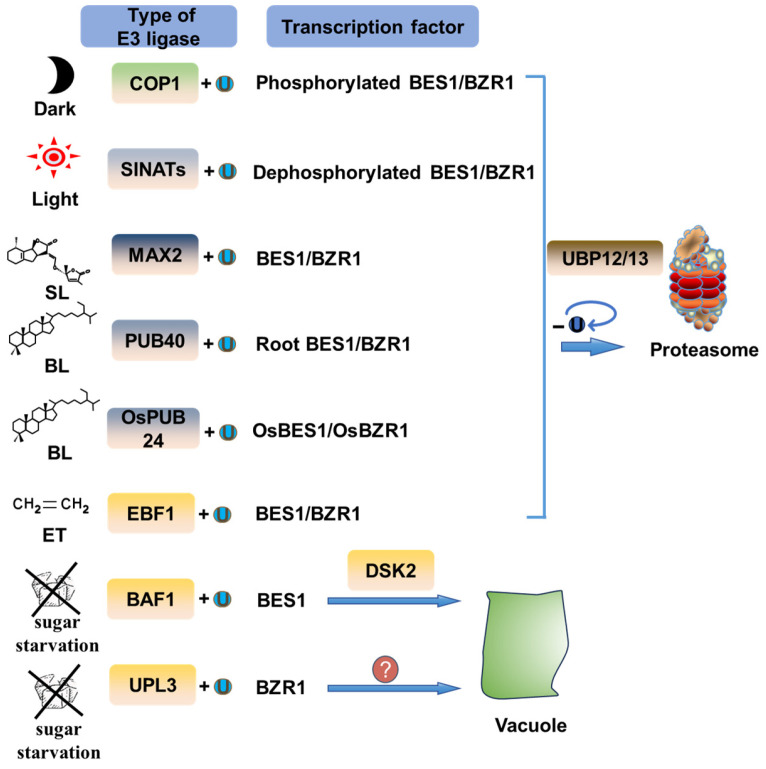
A current model for the regulation of BES1/BZR1 degradation by ubiquitination. The diagram illustrates that different ubiquitin ligases are induced by various conditions, leading to the ubiquitination and degradation of different states of BES1/BZR1 through distinct pathways. Under dark conditions, COP1 ubiquitinates the phosphorylated BES1/BZR1 for degradation, while under light conditions, SINATs ubiquitinate the dephosphorylated BES1/BZR1 for degradation. SL induces MAX2 to ubiquitinate BES1/BZR1, and BL in *Arabidopsis* induces PUB40 to specifically ubiquitinate BES1/BZR1 in roots, while in rice, it induces OsPUB24 to ubiquitinate BES1/BZR1. Ethylene induces EBF1 to ubiquitinate BES1/BZR1. Deubiquitination of BES1/BZR1 is mediated by UBP12/UBP13. Under sugar starvation, BAF1 ubiquitinates BES1, specifically binding to the autophagy receptor DSK2, leading to degradation within the vacuole through selective autophagy. Under sugar starvation conditions, UPL3 affects BZR1 ubiquitination and autophagy degradation. In the picture, “U” means ubiquitination.

The stability of BES1/BZR1 proteins is closely related to BR signaling and mainly depends on their phosphorylation status. Phosphorylated BES1/BZR1 exhibit relatively lower stability, whereas mutating phosphorylation sites can enhance their stability [95]. Subsequent studies revealed that the reduced stability of phosphorylated BES1/BZR1 is due to their susceptibility to ubiquitin-mediated degradation [112] (Figure 4). However, transcriptionally active dephosphorylated BES1/BZR1 also undergoes ubiquitin-mediated degradation [112].

### 4.1. Ubiquitin-Mediated Degradation of BES1/BZR1 by the Proteasome Pathway

Several ubiquitin ligases involved in hormone signaling have been identified to ubiquitinate and degrade BZR1 and BES1, thereby modulating BR signaling output and regulating plant development. Phytohormones orchestrate BES1/BZR1 ubiquitination. The strigolactone (SL)-activated F-box family ubiquitin ligase MAX2 polyubiquitylates BES1 in a phosphorylation-independent manner, targeting it for proteasomal degradation and thereby suppressing shoot branching [47]. Interestingly, MAX2-mediated ubiquitination of BES1 promotes its degradation, leading to the suppression of the transcription factor Branched 1 (BRC1) within the SL signaling pathway [113]. Collectively, this demonstrates how single ubiquitination events can coordinate multi-tiered signaling outputs. Another F-box ubiquitin ligase, Ethylene-Insensitive 3 Binding F-BOX Protein 1 (EBF1), serves as a negative regulator in the ethylene signaling pathway by targeting BZR1 for proteasomal degradation within the nucleus [48]. Additionally, EBF1 and its homolog EBF2 promote the degradation of Ethylene-Insensitive 3 (EIN3), which interacts with BZR1 to impede the formation of the seedling apical hook [114]. Under BR signal induction, the plant U-BOX family ubiquitin ligase PUB40 specifically degrades BZR1 in root cells in response to inorganic phosphate deficiency, thereby modulating root growth [49]. Since the study employed a 35S promoter, the possibility of similar regulatory mechanisms occurring above ground cannot be entirely excluded. This ubiquitination-based regulatory mechanism is evolutionarily conserved in rice, where OsPUB24 similarly targets OsBZR1 for degradation, thereby suppressing seedling vigor and leaf erection [50]. Without using a 35s promoter, the homologous OsPUB24 pathway shows less clear spatial regulation [50]. The regulation of BES1/BZR1 protein stability thus fosters coordinated interactions among these hormones.

Light-responsive E3 ubiquitin ligases control BES1/BZR1 stability. This regulation shows clear spatial patterns. COP1 targets phosphorylated BES1/BZR1 for ubiquitination, while Seven-IN-Absentia of *Arabidopsis thaliana* (SINATs) targets non-phosphorylated BES1/BZR1 for ubiquitination [51,52]. In *Arabidopsis*, there are five members of the SINATs family [115]. Except for SINAT5, which lacks the RING domain, the other four members can ubiquitinate non-phosphorylated BES1/BZR1 [52]. These RING family ubiquitin ligases jointly regulate the stability of the BES1/BZR1 protein, thereby affecting hypocotyl development [52]. Light conditions determine which E3 ligase dominates. Under dark conditions, COP1 predominantly ubiquitinates phosphorylated BES1/BZR1 in cotyledons to promote their degradation, while SINAT2-mediated ubiquitination of non-phosphorylated forms is inhibited in hypocotyls, thereby stabilizing BES1/BZR1 to drive hypocotyl elongation [51,52]. Light perception through phytochromes reverses this dynamic. SINATs enhance the degradation of non-phosphorylated BES1/BZR1 in hypocotyls to restrict growth, whereas COP1 can specifically regulate cotyledon growth by ubiquitinating phosphorylated BES1/BZR1 [51]. Under unsuitable temperature conditions, COP1 accelerates BES1 ubiquitination in cotyledon nuclei while attenuating this process in hypocotyl nuclei [116]. This spatiotemporal regulation promotes hypocotyl elongation through BES1 stabilization and inhibits cotyledon growth by proteasomal degradation, thereby demonstrating the complexity of tissue-specific BES1/BZR1 ubiquitination regulation to adapt to environmental changes [116]. Additionally, Blade On-Petiole-1 (BOP1), a component of the CUL3 ubiquitin ligase complex, destabilizes PIF4 to indirectly modulate the stability of BES1/BZR1 [117,118]. The specific composition of the ubiquitin ligase complex containing BOP1 that regulates BES1/BZR1 remains to be elucidated, and the precise molecular mechanisms underlying this ubiquitination-mediated regulation are not yet fully understood.

The ubiquitination of BES1/BZR1 is dynamically modulated by other enzymes. Recent parallel investigations have elucidated the roles of the deubiquitinating enzyme UBP12/13 in regulating BES1/BZR1 from separate perspectives [53,54]. These studies revealed that UBP12/13 deubiquitinates BES1/BZR1 regardless of its phosphorylation status, thereby allowing both phosphorylated and dephosphorylated forms to undergo deubiquitination [53,54]. Furthermore, the SUMO ligase SIZ1 binds to the PEST domain of BES1, at its ubiquitin recognition site [55]. This binding potentially inhibits the ubiquitination of BES1 through competitive interactions, thereby enhancing its protein stability [55,119]. In parallel, the ubiquitin ligase Ring Zinc finger 1 (RZF1) regulates BEH3, a homologous protein of BES1/BZR1 with over 90% sequence similarity, affecting plant sensitivity to abscisic acid and osmotic stress [58]. However, direct biochemical evidence is still lacking to confirm whether RZF1 regulates the abscisic acid signaling pathway through the ubiquitination of BEH3, or if it can ubiquitinate BES1/BZR1.

### 4.2. Ubiquitination-Mediated Degradation of BES1/BZR1 via Autophagy

Compared to UPS-mediated degradation under normal conditions, stress can trigger the autophagic degradation of certain proteins to maintain cellular homeostasis [120]. As a dually regulated target, BES1/BZR1’s fate—whether undergoing proteasomal or autophagic degradation—is primarily determined by its cellular metabolic status [33,56]. Under dark-induced sugar starvation conditions, the inactivation of the Target of Rapamycin (TOR) kinase activates autophagy, leading to BES1/BZR1 degradation and subsequent inhibition of plant growth [33,121,122]. The autophagy of BES1 is mediated by the F-box family ubiquitin ligase BES1-ASSOCIATED F-BOX 1 (BAF1), which promotes the formation of K63 chains [56]. Mechanistically, BES1 binds the selective autophagy-specific receptor Dominant Suppressor of Kar 2 (DSK2), which initiates its autophagic degradation [33]. The activity of DSK2 is further regulated by BIN2, which phosphorylates DSK2 to enhance its interaction with autophagy-related 8 (ATG8), thus promoting the autophagy of BES1 [33]. This complex interaction underscores the sophisticated regulatory mechanisms governing BES1 ubiquitination. Notably, BAF1 is not an inducible expression protein under sugar starvation conditions; instead, under non-stress conditions, it collaborates with MAX2 and SINAT2 to ubiquitinate BES1 for proteasomal degradation [56]. However, the mechanisms by which BAF1 assists other ubiquitin ligases in forming K48 ubiquitination chains and its response to sugar starvation signals, which guide a shift in BES1 ubiquitination regulation, remain to be fully elucidated.

Although BES1 and BZR1 share high sequence similarity and are co-regulated by multiple proteins, their mechanisms of autophagy regulation are distinct. The selective autophagy receptor DSK2, which targets BES1 for degradation, does not induce the autophagy of BZR1 [33]. Recent studies have identified two ubiquitin ligases, BAF1 and Ubiquitin-Protein Ligase 3 (UPL3), that interact with BZR1 under glucose starvation conditions [57]. However, genetic evidence indicates that the loss of UPL3 reduces the ubiquitination-dependent autophagic degradation of BZR1 under sugar starvation conditions, whereas the loss of BAF1 does not exhibit this effect [57]. Genetic analyses demonstrate that UPL3 functions upstream in the regulatory hierarchy to mediate BZR1 ubiquitination and subsequent autophagic degradation [57]. However, direct experimental evidence to support this hypothesis is still lacking. Considering that HECT domains’ promiscuity in animals is known to ubiquitinate a broad range of autophagy-related proteins in animals, UPL3 might regulate the autophagic degradation of BZR1 by targeting proteins associated with its autophagy pathway [123]. Additionally, it should be noted that UBP12/13 can simultaneously deubiquitinate both the K48 and K63 linkages of BES1/BZR1 [53,54]. However, whether UBP12/13 exhibits preference for specific types of ubiquitin chains remains controversial. As a common effector in these two degradation pathways, they may play a regulatory role in determining the degradation route of BES1/BZR1. These findings suggest we need to consider BES1/BZR1 ubiquitination-mediated degradation in a more complex multidimensional context.

## 5. Ubiquitination Regulation of Other BR Signal Transduction Components

In addition to the critical regulatory components BRI1, BIN2, and BES1/BZR1, BR signal transduction involves a complex network of over 200 proteins, many of which are regulated by ubiquitination [124,125]. RLCKs, a group of protein kinases typically localized in the cytoplasm, are often associated with the plasma membrane through post-translational lipid modifications or interactions with membrane-associated proteins [126]. Notably, specific RLCKs involved in BR signaling are also subject to ubiquitination-mediated regulation. For example, BKI1 inhibits BRI1 kinase activity in *Arabidopsis*, but this inhibition is counteracted by PUB30, a member of the PUB family [59]. PUB30 promotes BKI1 ubiquitination and subsequent degradation via the UPS, thereby releasing BRI1 from inhibition [59]. In wheat, a homolog of BKI1 is ubiquitinated by the RING-type E3 ubiquitin ligase TaZnF-B, leading to its proteasomal degradation via the UPS [60]. The species-specific regulation of BKI1 homologs by distinct E3 ligases suggests evolutionary divergence in BR signaling mechanisms across different organisms. Another RLCK, Botrytis-Induced Kinase1 (BIK1), negatively regulates BR signal transduction. In the presence of BR, BIK1 is phosphorylated by BRI1, leading to its dissociation [127]. However, BIK1 demonstrates contrasting functionality in plant immunity, where it positively regulates defense signaling through association with the BAK1 co-receptor rather than BRI1 [128,129]. The ubiquitination dynamics of BIK1 are tightly regulated by the phosphorylation status and E3 ligases. Under normal conditions, non-phosphorylated BIK1 interacts with PUB2/4, which facilitates its ubiquitination and degradation [62]. Immune signal perception triggers BIK1 phosphorylation and subsequent ubiquitination by PUB25 and PUB26, directing it toward proteasomal degradation [61]. Phosphorylation status determines ligase specificity—RHA3A/RHA3B target hyperphosphorylated BIK1 [63], whereas RING-domain ligase 1 (RGLG1) and RGLG2 target hypo-phosphorylated forms [64]. Additionally, RGLG2 can inhibit PUB25’s ubiquitin ligase activity, while PUB25 mediates RGLG2’s proteasomal degradation [64]. Key unresolved questions remain regarding the intersection of these regulatory mechanisms. The differential phosphorylation states induced by BRI1 (BR signaling) versus BAK1 (immunity signaling), combined with distinct E3 ligase partnerships, establish a molecular basis for BR immunity crosstalk. Determining whether BIK1-associated ubiquitin ligases involved in immune responses also influence BR signal transduction represents an important area for future investigation.

## 6. Conclusions and Remarks

Ongoing research on ubiquitination continues to deepen our understanding of how UPS dynamics regulate substrate degradation and interface with BR signaling networks. By developing real-time monitoring systems (e.g., UbiReal), future studies can precisely capture the spatiotemporal regulation of ubiquitination. To decipher multi-pathway crosstalk, integrating CRISPR screening with multi-omics analyses will systematically map interactions among UPS-mediated degradation, endocytic trafficking, and autophagic pathways. Additionally, the interaction between substrates and specific E3 ligases in response to their local environments can lead to distinct degradation fates. While ubiquitination’s role in regulating BR signaling has been characterized in *Arabidopsis*, its functional implications have not yet been systematically elucidated in other plant species, particularly in food crops. Considering the evolutionary divergence in BR synthesis, metabolism, and signaling pathways between monocotyledons (e.g., cereals) and dicotyledons, systematic efforts leveraging pan-genome association studies of E3 ligase variants are needed to identify essential ubiquitination-related components regulating BR signaling in grass species. A comprehensive analysis of the molecular mechanisms linking ubiquitination to growth regulation and stress adaptation, particularly through engineered ubiquitin variants (UbVs) that selectively perturb pathway crosstalk, will represent a critical frontier in BR biology.

In summary, to advance our understanding of ubiquitin-mediated BR signaling regulation, future investigations should focus on the following: (1) precise mapping of ubiquitin chain topology (e.g., K48 vs. K63 linkages) using nanobody-based biosensors and characterizing their distinct functional implications in receptor complex dynamics for the predictive modeling of BR response modules, (2) spatiotemporal analysis of degradation pathway crosstalk under varying physiological contexts via microfluidic-based root phenotyping platforms that integrate UPS/autophagy reporters, and (3) comparative studies across phylogenetically diverse species employing synthetic biology approaches (e.g., orthologous E3 swapping between maize and *Arabidopsis*) to elucidate evolutionary mechanisms underlying ubiquitination-mediated integration of environmental signals with developmental programming. Systematic investigation of these research priorities will elucidate both conserved mechanisms and taxon-specific adaptations in ubiquitin-mediated brassinosteroid signaling regulation. These fundamental insights will provide a molecular framework for developing precision breeding strategies that optimize resource-use efficiency through targeted modulation of BR signaling pathways.

## Figures and Tables

**Table 1:** Ubiquitin-related proteins in the BR signaling pathway.

Functional Classification	Name	Gene ID	Protein Family	References
BRI1 de/ubiquitination	UBC35	AT1G78870	UBC	[36]
	UBC36	AT1G16890	UBC	[36]
	PUB12	AT2G28830	U-BOX	[23]
	PUB13	AT3G46510	U-BOX	[23]
	UBP12	AT5G06600	DUB	[37]
	UBP13	AT3G11910	DUB	[37]
	OsELT1	Os02g58390	RLK	[38]
	OsSMG3	Os03g03080	UBC	[39]
	OsDGS1	Os03g07069	RING	[39]
	SiUBC32	Seita.9G428900	UBC	[40]
	SiSGD1	Seita.9G123200	RING	[40]
	TASH3	AT2G07360	T-PLATE complex	[41]
	AMSH3	AT1G15130	DUB	[42]
	BIL4	AT3G63310		[43]
BIN2/OsGSK2 ubiquitination	KIB1	AT4G12810	F-BOX	[44]
	OsTUD1	Os03g13010	U-BOX	[45]
	BRFP1	AT2G45100	F-BOX	[46]
	BRFP2	AT3G09360	F-BOX	[46]
BES1/BZR1 de/ubiquitination	MAX2	AT2G42620	F-BOX	[47]
	EBF1	AT2G25490	F-BOX	[48]
	PUB40	AT5G40140	U-BOX	[49]
	OsPUB24	Os03g06571	U-BOX	[50]
	COP1	AT2G32950	RING	[51]
	SINATs	AT3G58040	RING	[52]
	UBP12	AT5G06600	DUB	[53,54]
	UBP13	AT3G11910	DUB	[53,54]
	SIZ1	AT5G60410	SUMO E3 ligase	[55]
	DSK2	AT2G17200	Autophagy receptor	[33]
	BAF1	AT1G76920	F-BOX	[56]
	UPL3	AT4G38600	HECT	[57]
BEH3 ubiquitination	RZF1	AT3G56580	RING	[58]
BKI1 ubiquitination	PUB30	AT3G49810	U-BOX	[59]
	TaZnF-B	TraesCS4B02G042900	RING	[60]
BIK1 ubiquitination	PUB25	AT3G19380	U-BOX	[61]
	PUB26	AT1G49780	U-BOX	[61]
	PUB2	AT5G67340	U-BOX	[62]
	PUB4	AT2G23140	U-BOX	[62]
	RHA3A	AT2G17450	RING	[63]
	RHA3B	AT4G35480	RING	[63]
	RGLG1	AT3G01650	RING	[64]
	RGLG2	AT5G14420	RING	[64]

## Data Availability

Not applicable.

## Abbreviations

The following abbreviations are used in this manuscript:

ALIX	apoptosis-linked gene 2-interacting protein X
APC/C	Anaphase-Promoting Complex/Cyclosome
ATG8	autophagy-related 8
BAF1	BES1-ASSOCIATED F-BOX 1
BAK1	BRI1-Associated Receptor Kinase 1
BES1	bri1-EMS suppressor 1
BIK1	Botrytis-Induced Kinase 1
BIL4	Brassinazole-Insensitive-Long hypocotyl 4
BIN2	Brassinosteroid-Insensitive 2
BKI1	BRI1 Kinase Inhibitor 1
BOP1	Blade On-Petiole-1
BRC1	Branched 1
BRFP1	BRASSINOSTEROID F-BOX Protein 1
BRI1	Brassinosteroid-Insensitive 1
BRs	Brassinosteroids
BSKs	BR Signaling Kinases
BSL1-3	BSU1-like proteins 1–3
BSU1	bri1 suppressor 1
BZR1	Brassinazole-Resistant 1
CDG	Constitutive Differential Growth
CIE	clathrin-independent endocytosis
CLASP	cytoplasmic linker protein-associated protein
CME	clathrin-mediated endocytosis
COP1/SPA1	Constitutively Photomorphogenic 1/Suppressor of phyA-105
CRLs	cullin-RING ligases
CUL1	Cullin 1
DGS1	grain size 1
DSK2	Dominant Suppressor Of Kar 2
DUBs	Deubiquitinating enzymes
EBF1	Ethylene-Insensitive 3 Binding F-BOX Protein 1
EIN3	Ethylene-Insensitive 3
ENTH	Epsin N-Terminal Homology
ER	endoplasmic reticulum
ERAD	ER-associated degradation
ESCRT	endosomal sorting complex required for transport
HECT	E6AP carboxyl terminus
KIB1	Kink suppressed in bzr1-1D 1
LRR	leucine-rich repeat
MAX2	More Axillary Growth Locus 2
MVB	multivesicular bodies
PIF3	Photoperiod-Interactive Factor 3
PM	plasma membrane
PP2A	protein phosphatase 2A
PPKLs	protein phosphatases with kelch-like
RGLG1	RING-domain ligase 1
RHA3A	RING-H2 Finger A3A
RING	Really Interesting New Gene
RLCKs	receptor-like cytoplasmic kinases
RZF1	Ring Zinc finger 1
SGD1	small grain and dwarf 1
SINATs	Seven-IN-Absentia of *Arabidopsis thaliana*
SL	strigolactone
TGN/EE	trans-Golgi network/early endosome
TOR	target of rapamycin
TPC	TPLATE complex
TUD1	Taihu Dwarf1
UPL3	Ubiquitin-Protein Ligase 3
UPS	ubiquitin–proteasome system
WSB1	WD repeat and SOCS box-containing protein 1
